# Investigating sensitivity to multi-domain prediction errors in chronic auditory phantom perception

**DOI:** 10.1038/s41598-024-61045-y

**Published:** 2024-05-14

**Authors:** Anusha Yasoda-Mohan, Jocelyn Faubert, Jan Ost, Juri D. Kropotov, Sven Vanneste

**Affiliations:** 1 Lab for Clinical and Integrative Neuroscience, School of Psychology, Trinity College Institute for Neuroscience, Trinity College Dublin, College Green, Dublin 2, Ireland; 2 Global Brain Health Institute, Trinity College Dublin, Dublin 2, Ireland; 3 Faubert Lab, School of Optometry, University of Montreal, Montreal, Canada; 4 Brain Research Center for Advanced International Innovative and Interdisciplinary Neuromodulation, Ghent, Belgium; 5 N.P. Bechtereva Institute of the Human Brain of Russian Academy of Sciences, St. Petersburg, Russia

**Keywords:** Tinnitus, Predictive coding, Sensory prediction error, Visual domain, Deafferentation, Diagnostic markers, Neurological disorders

## Abstract

The perception of a continuous phantom in a sensory domain in the absence of an external stimulus is explained as a maladaptive compensation of aberrant predictive coding, a proposed unified theory of brain functioning. If this were true, these changes would occur not only in the domain of the phantom percept but in other sensory domains as well. We confirm this hypothesis by using tinnitus (continuous phantom sound) as a model and probe the predictive coding mechanism using the established local–global oddball paradigm in both the auditory and visual domains. We observe that tinnitus patients are sensitive to changes in predictive coding not only in the auditory but also in the visual domain. We report changes in well-established components of event-related EEG such as the mismatch negativity. Furthermore, deviations in stimulus characteristics were correlated with the subjective tinnitus distress. These results provide an empirical confirmation that aberrant perceptions are a symptom of a higher-order systemic disorder transcending the domain of the percept.

## Introduction

The predictive coding model is now widely gaining recognition and consensus to explain the integrated functioning of the brain. The theory is that the brain maintains an internal prediction model with which it compares the incoming sensory information to produce its perception of the environment around it^1–3^. The difference between the prediction and the input is called a prediction error^2^. If the error is large and strong, it modifies the internal model by changing the prediction and thereby the percept^2^.

Perceptual disorders in different sensory domains such as tinnitus (a phantom ringing in the ears)^4^, chronic pain^5^, phantom smell^6^ and taste^7^, Charles Bonet syndrome^8^ where patients perceive phantom images that are illogically related to the surrounding and other hallucinatory experiences are discussed as a maladaptive compensation of an aberration in the brain’s predictive coding system^3^. This aberration is most commonly brought on by loss of sensory information in the domain of the phantom percept—hearing loss^9^, damage to peripheral nerve endings in the extremities^10^, sensorineural loss in the olfactory or gustatory system^11^, macular degeneration^12^ etc. respectively. Although the deafferentation is limited to the sensory domain of the phantom percept, predictive coding is a much broader, higher-order (systems-level or systemic) process that controls all the senses. So, the question now arises if the maladaptive predictive processing of the brain in any of these disorders is just limited to the domain of deafferentation or if it is a symptom of a disorder in higher-order systemic processing that extends to other sensory domains as well.

In the current study, we use tinnitus as a model and probe the predictive coding system in the sensory domain of the percept and deafferentation (auditory) and a domain with no phantom percept and functionally more intact (visual). Tinnitus is a simple auditory phantom percept which is rarely accompanied by symptoms of psychosis^13^. The predictive coding model of tinnitus is comprehensively discussed by several researchers in the field and attempts to provide a universal and integrated explanation to the generation of a very heterogenous disorder^2,3,14,15^. One of the hypothesis is that there is a tinnitus precursor signal which becomes more precise and strong in the presence of hearing loss or other changes in the bottom-up pathways^15^. If the strength of the precursor is not minimised by the top-down inhibitory system, the strong tinnitus precursor signal can override the current prediction of perceiving silence in the absence of an external sound source and updating it to perceiving tinnitus^15^. Chronic stress can also change our bodily response to day-to-day events^16^ there by changing the context in which input is processed. Stress shown to play an important role in tinnitus generation^17^, even in the absence of hearing loss. With time, the brain learns that perceiving tinnitus may be the new normal leading to a chronic tinnitus percept that is difficult to modify^15^. The empirical evidence for tinnitus being a disorder of predictive coding is becoming more concrete^18,19^. In a recent study, we showed that tinnitus patients were more sensitive to auditory prediction errors^20^ encoded by the previously well-established local–global oddball paradigm^21–23^. We are particularly interested if this maladaptive predictive processing in tinnitus surpasses the auditory domain to reflect changes in other sensory domains as well.

The current study is designed as a pilot study in a clinical setting. It uses the auditory (similar to the recent study^20^) and an equivalent visual adaptation of the local–global oddball paradigm to probe the predictive coding system of the brain and investigate maladaptive changes in tinnitus compared to non-tinnitus participants. Here we investigate two types of prediction errors—sensory and contextual—based on whether we change the *input* to the model or the *prediction* of the model. These prediction errors are empirically quantified using evoked response potential (ERPs) corresponding to the different stimulus sequences which are recorded using EEG. Subtracting the ERP of the frequent (standard, expected omission) from that of the rare (deviant, unexpected omission) sequences will generate a prediction error signal which has two signature components—(1) mismatch negativity (MMN) and (2) late positive potential popularly known as the P300^24,25^.

The MMN was first discovered in the auditory domain as a negative going potential between 100 and 250 ms post-stimulus onset in the frontal electrodes that encodes an automatic deviance in stimulus characteristics^24^. This has been explained under both the predictive coding framework^26–28^ and also under the neural adaptation framework where the change reflects a deviation from the adaptation of the neural groups to repetitive stimuli^29,30^. The MMN was later also identified in the visual domain^31^. In the context of the difference between the two omission sequences, the MMN becomes an early omission response capturing the pre-attentive, hierarchical predictive process^23^.

The P300 is an attention-driven component which is a positive going potential 250 ms post-stimulus onset^23,25^ that requires the participant to engage in the task actively or passively. In a three-stimulus oddball paradigm, where there are two rare stimuli with different probabilities, the more deviant stimulus shows a positive going potential in the fronto-central electrodes marking a shift in attention and the less deviant stimulus shows a positive going potential in the parietal electrodes^20,24,32^. A third component that is being explored in the predictive coding ERP space is the anticipatory pre-stimulus signal that records the neural signature of the expectation of the upcoming input^18^.

Based on these previous findings, we hypothesise that if tinnitus were purely an auditory predictive processing disorder, we would expect to observe a significant difference between the tinnitus and control group in the prediction errors produced by sensory and contextual changes in the incoming stimuli during the MMN and P300 time frames only in the auditory but not in the visual domain. However, if tinnitus were higher-order systemic problem, these differences would transcend its domain of deafferentation (auditory) and be reflected in another sensory domain that is independent of it (visual) as well. Given the mechanistic similarity between tinnitus and other phantom percepts, showing that phantom percepts present changes in predictive coding reflected in sensory systems other than the domain of the percept would be a pivotal finding in understanding the neural mechanism underlying different perceptual disorders.

## Materials and methods

The current study used the well-established local–global oddball paradigm and was implemented in both the auditory and visual domains. The participants consisted of those with and without tinnitus. In the effort to balance running time of the experiment, clinical evaluations of patients and capturing the holistic picture of prediction errors in the auditory and visual domains, we reduced the number of blocks in this experiment as compared to the original experiment. The study consisted of 4 blocks of auditory and visual stimulation with the experiment lasting up to an hour with a self-timed break in between each block.

### Ethical statement

The study was approved by the ethical committee at Trinity College Dublin and was in accordance with the Declaration of Helsinki. An explicit informed consent to participate in the study was obtained from all participants. Tinnitus participants were patients who visited the Brain Research center for Advanced Innovative & Interdisciplinary Neuromodulation (Brai3n) clinic in Ghent, Belgium. Control participants were volunteers from the clinic and the community.

### Participants

The study consists of 16 participants with tinnitus (*M* = 47.87 years, *SD* = 15.04 years; 10 males, 6 females) and 13 participants without tinnitus (*M* = 42.85 years, *SD* = 16.14 years; 5 males and 8 females). The study was powered based on a recently published study^33^. The two groups were matched for age (*t*(27) = 0.87, *p* = 0.394, d = 0.32), gender(χ^2^ = 1.66, *p* = 0.197) and overall audiogram (*F*(3.34,60.68) = 0.822, *p* = 0.497, partial η^2^ = 0.043). These were determined using an independent samples *t*-test, a chi-square test and a repeated measures ANOVA with groups (tinnitus and control) as between-subjects variable, frequencies (explained below in the audiogram section) and side (left and right) as repeated measures. Exclusion criteria included Meniere’s disease, chronic ear infections, otosclerosis, tumors, mental disorders and chronic eye disorders. All participants either had normal or corrected-to-normal vision. Patients presented with tinnitus anywhere between 3 months and 20 years.

### Audiological and behavioural measures

**Figure 1:**
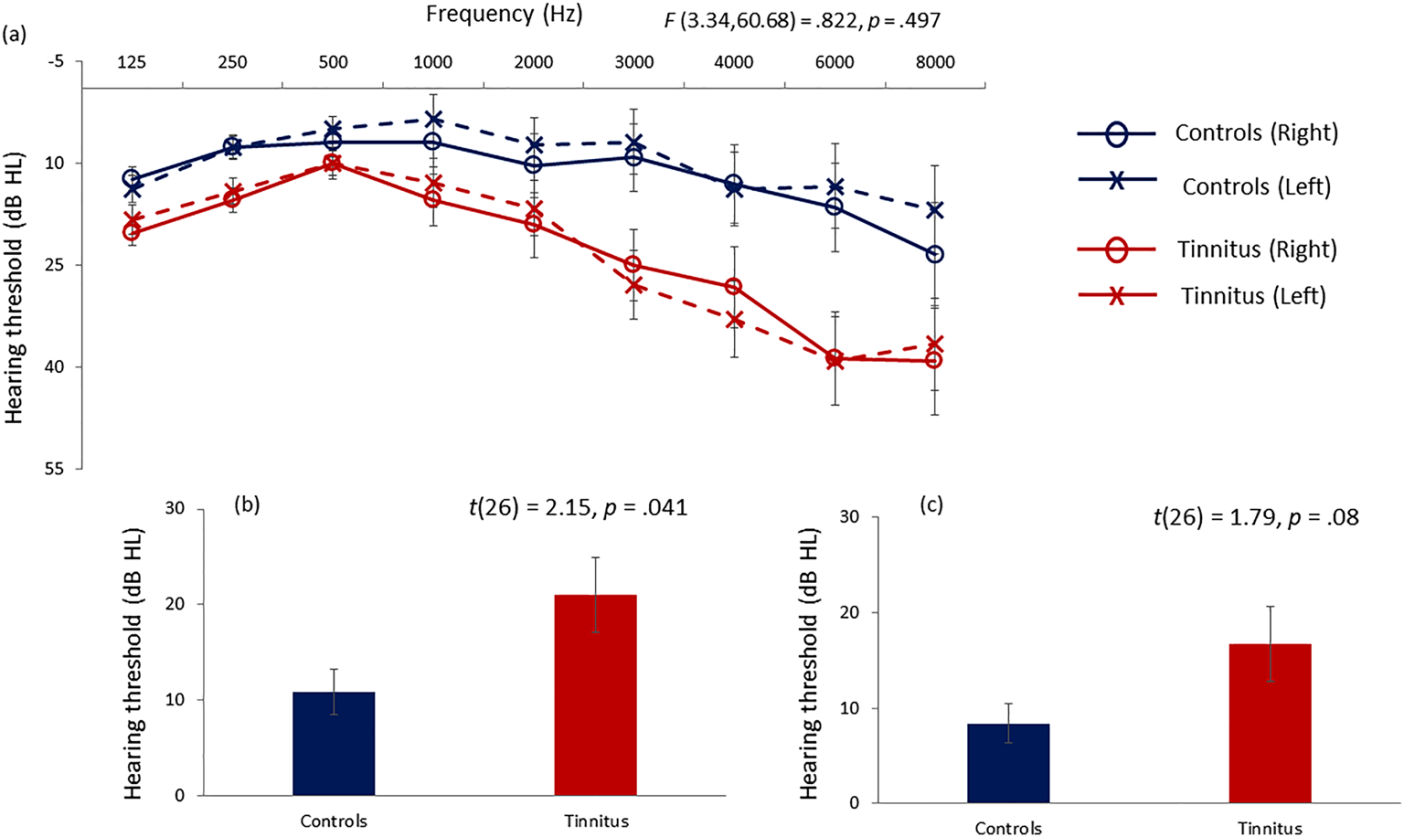
Hearing assessment in the tinnitus and control groups. (**a**) The pure tone audiogram thresholds at the different frequencies are shown for the controls (blue) and the tinnitus (groups). (**b**) The mean hearing loss calculated over all the frequencies, and (**c**) the mean hearing disability calculated for 500, 1000, 2000, 4000 Hz are displayed for the controls (blue) and tinnitus (red) groups. The error bars represent standard error.

All participants underwent audiological measurements which included a pure tone audiogram testing the threshold of hearing at frequencies 125, 250, 500, 1000, 2000, 3000, 4000, 6000, and 8000 Hz in both left and right ears according to the procedures prescribed by the British Society of Audiology. The overall audiogram of the participants from the two groups were matched as indicated above with the two groups showing a significant difference in the mean hearing loss calculated as the mean of the audiological thresholds at all frequencies in both ears (*t*(26) = 2.15, *p* = 0.041, d = 0.83) and a trending difference between the two groups for the measure of hearing disability measured at speech frequencies which is the mean of the hearing thresholds at 500, 1000, 2000 and 4000 Hz (*t*(26) = 1.79, *p* = 0.08, d = 0.69) as used by the World Health Organisation^34^. This is particularly important given that the auditory stimulus is centered at 500 Hz. The pure tone audiogram, mean hearing loss and hearing disability thresholds for the two groups are displayed in Fig. 1.

The tinnitus characteristics such as the loudness and distress were measured using self-report questionnaires. In addition, the tinnitus type (10 patients with pure tone, 2 patients with noise-like and 3 patients with a combination, 1 missing value) and laterality (3 patients with left-sided tinnitus, 2 patients with right-sided tinnitus, 5 patients with tinnitus on both ears equally, 4 patients with tinnitus on both ears but with dominant left-sided tinnitus and no one with tinnitus on both ear with a dominant right-sided tinnitus, 2 missing values) were also recorded to understand the heterogeneity of the group. Tinnitus loudness and distress were measured using a visual analogue scale (VAS) for loudness (*M* = 5.24, *SD* = 2.39) and distress (*M* = 4.39, *SD* = 2.51). VAS is a 10-cm scale with end points at a 0 = “no tinnitus”/”no distress” and 10 = “as loud as imaginable”/“suicidal levels of distress”. Participants also completed a self-report questionnaire for Tinnitus Handicap Inventory (THI) (*M* = 49.12, *SD* = 20.24) which is designed to assess the tinnitus-related distress and impact on their lives. Most participants filled out the Dutch-translated versions of the questionnaires except a few who opted for the English versions.

### Event-related potential (ERP) study using an oddball paradigm

**Figure 2:**
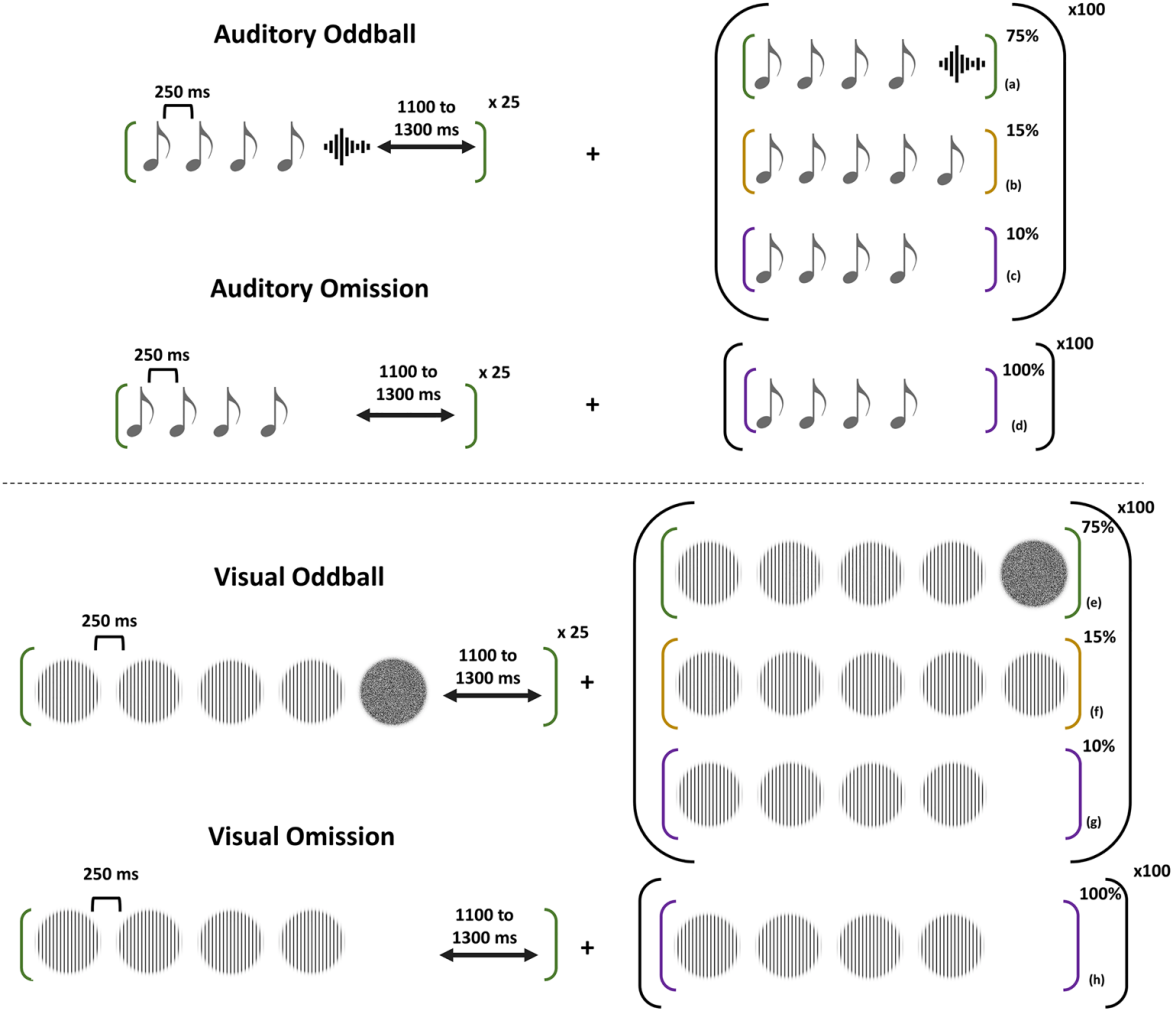
A visual representation of the local–global oddball paradigm adapted to the auditory and visual domain. The top section represents the auditory paradigm where each tone represents a 500 Hz pure tone and the signal represents a white noise burst. The bottom section represents the visual paradigm where each sinusoidal grating is designed at 3 cpd and the noise stimulus is a white noise. In both paradigms the standard is played 25 times to establish the global rule and standard-deviant-omission sequences are played at 75%–15%–10% probability. The omission sequence is also played separately at 100%.

The main study encapsulates the use of EEG to record brain activity in response to auditory and visual stimuli that are played in a specific paradigm developed by Wacongne and colleagues^23^. The auditory segment consisted of three different stimulus sequences as shown in Fig. 2 presented in different probabilities. The standard sequence consisted of four 500 Hz tones followed by one white noise burst; the deviant sequence consisted of five 500 Hz tones and the omission sequence consisted of four 500 Hz tones and a silence in the place of the 5th stimulus. Each stimulus was 50 ms in duration with a 7 ms rise-fall time separated by 250 ms. The sequences were matched to have the same RMS power. This particular paradigm was chosen based on the results of a recent paper^33^. The sounds were played binaurally through stereo headphones.

All stimuli were played at the most comfortable loudness level following the procedure of the recently published study^33^. This was determined by presenting the sequence with five tones at varying loudness levels going from full volume on the headphones (84 dB SPL) in 5 dB attenuation steps until it was 40 dB softer than the original sound and then increased in 5 dB attenuation steps back to full volume. For every sequence the participant was asked to rate the loudness on a scale of 1–7, where 7 = “uncomfortably loud” and 1 = “very soft”. 4 was considered most comfortable loudness. For those patients who were uncomfortable going through the procedure since some of the sounds could be uncomfortably loud, we played some of the softer sounds and let them choose what level they wanted to hear. The standard sequence of the 4 tones followed by noise burst was chosen at the same attenuation level as the level picked by the participant.

The visual equivalent of this paradigm consisted of 2 cycles per degree (cpd) sinusoidal gratings and a visual noise. 2 cpd was used because this falls under the low-mid frequency range just like the auditory stimuli. The standard visual sequence consisted of four sinusoidal gratings followed by one visual noise stimulus (2 × 2 pixel binary noise); the deviant sequence was five sinusoidal gratings; the omission sequence was 4 sinusoidal gratings followed by a blank screen where the stimulus should’ve been. All stimuli were 100% contrast played on a grey mean luminance background (49.58 cd/m^2^). The luminance output was gamma corrected and we used the ‘Noisy Bits’ method for producing a continuous luminance resolution^35^. The image dimensions were 1440 × 900 pixels with a bit depth of 24. The sinusoidal gratings of 900 pixels in diameter was placed at the center of the image. The participants sat at 171 cm from the screen. The stimuli were played on a 21″ LED Dell Monitor.

The auditory and visual paradigms were played in four blocks—three blocks of oddball presentation and one block consisting solely of omission sequences. Each block consisted of 125 trials. In the oddball blocks, 25 trials of the standard were first presented followed by 100 trials of standard, deviant and omission sequences pseudo randomly presented at 75%, 15% and 10% probability respectively so that no two similar type of deviants were presented continuously and there were at least 2 standard between similar deviants. The omission block consisted of 125 trials of omission sequences. The inter-trial interval was jittered between 1400 and 1600 ms. This resulted in 300 standard, 45 deviant, 30 unexpected omission and 125 expected omission trials. The order of presentation was randomised between the visual and auditory paradigms and between the oddball and omission blocks in each paradigm. A short break was given between each block and between each paradigm.

### EEG data collection and pre-processing

The stimuli were presented using PsyTask which was controlled by the WinEEG software. The EEG was sampled using Mitsar 201 amplifiers at 250 Hz. The data was collected using a 19 channel Mitsar EEG cap designed using the International 10–20 placement system. The data was band-pass filtered online from 0.1 to 70 Hz and was collected with a reference close to Cz. Offline, the data was re-referenced to an average reference, filtered between 0.55 and 45 Hz, cleaned for artifacts such as eyeblinks, saccades, muscle movements etc. using a temporal independent component analysis (ICA) using infomax algorithm and epoched between − 400 and + 2352 ms relative to the onset of the first stimulus of the sequence. The data was further manually inspected for epochs with large deviations and were cleaned before comparing the two groups. A multivariate ANOVA to test the difference between the number of ICA components remaining in the two groups and two paradigms after cleaning reveal no significant differences (*F* (2,26) = 1.14, *p* = 0.334, partial ƞ^2^ = 0.08).

### ERP post processing and analysis

The prediction error is defined as the difference between the incoming sensory input and the existing prediction^2^. When auditory and visual stimuli are presented in an oddball paradigm, the frequent sequence creates a sensory memory trace which acts as a prediction or expectation of the incoming input. The rare sequence creates a deviance from the expectation and the difference between the two can be regarded as the prediction error. The prediction error signal was calculated from the averaged ERPs from the single trial responses to each stimulus sequence resulting in three different conditions in both the auditory and visual paradigms for all participants: (1) deviant (–) standard; (2) unexpected omission (–) standard; (3) unexpected omission (–) expected omission. The first two prediction errors represent a deviance in the expectation of the sensory information of the 5th stimulus and represent a prediction error that is driven by changes in stimulus characteristics. In (3), the stimulus characteristics of the expected and unexpected stimuli are the same, but the difference between these stimuli is purely a difference in expectation and can be conceived as a prediction error driven by changes in contextual information.

Given that there is a difference between the number of trials in the standard, deviant, expected and unexpected omission sequences, the frequent stimuli were randomly chosen from the available number to match the number of rare stimuli for each person, in each condition mentioned above. The average ERP was calculated across the responses of the both the stimulus sequences for all conditions and then subtracted as indicated above. This was repeated 100 times for each participant in both groups and each prediction error condition to create a dataset of trials representing the sensory and contextual prediction errors for each individual. The prediction error responses were compared between the two groups using a non-parametric cluster-based comparison in FieldTrip^36^ where a between-trials comparison was performed comparing all the trials of each participant from the control group with each participant of the tinnitus group using the dataset of trials for each individual. This resulted in a total of 208 cluster maps (13 × 16 comparisons) each corrected at the cluster-level (alpha = 0.05). This cluster was formed keeping 2 neighbouring channels around each channel. The neighbours for each channel was determined using the neighbours matrix suggested by FieldTrip for a 19-channel electrode placement. The results represent the clusters that survived in over 92.5% of these comparisons. Keeping in mind the pilot nature of the study, we restrict the cut-off at 92.5%.

The topographical plots were plotted for the mean of the time points that showed a significant difference in the time frame of the MMN (up to 250 ms) and P300 (> 250 ms) and additionally in the pre-stimulus timeframe for the context-driven prediction error in both the auditory and visual paradigms. The cluster of electrodes were determined from the result of the ERP analysis based on which electrodes showed a significant difference in similar time points in the time frames mentioned above in over 92.5% of the comparisons. The ERP for the cluster of the electrodes was plotted with the time points showing the significant difference between the two groups in the each of the time frames analysed above.

### Post-hoc correlation with behavioural data

The mean amplitude of the significant channel-time point cluster averages in the MMN and timeframe in the auditory and visual paradigms was each correlated with the age and mean hearing loss combining the control and tinnitus groups together and with VAS for loudness, duration and the overall THI score in only the tinnitus group using Spearman rank correlation. The resulting correlations were corrected for multiple comparisons using the Benjamini-Hoschburg false discovery rate of alpha = 0.05.

### Ethical statement

An explicit informed consent was obtained from all participants of the study.

## Results

A general note is that all ERPs plotted in the figures represent a prediction error i.e. the difference between an unexpected and expected condition.

### Stimulus-driven prediction error: difference between deviant and standard stimulus sequences

**Figure 3:**
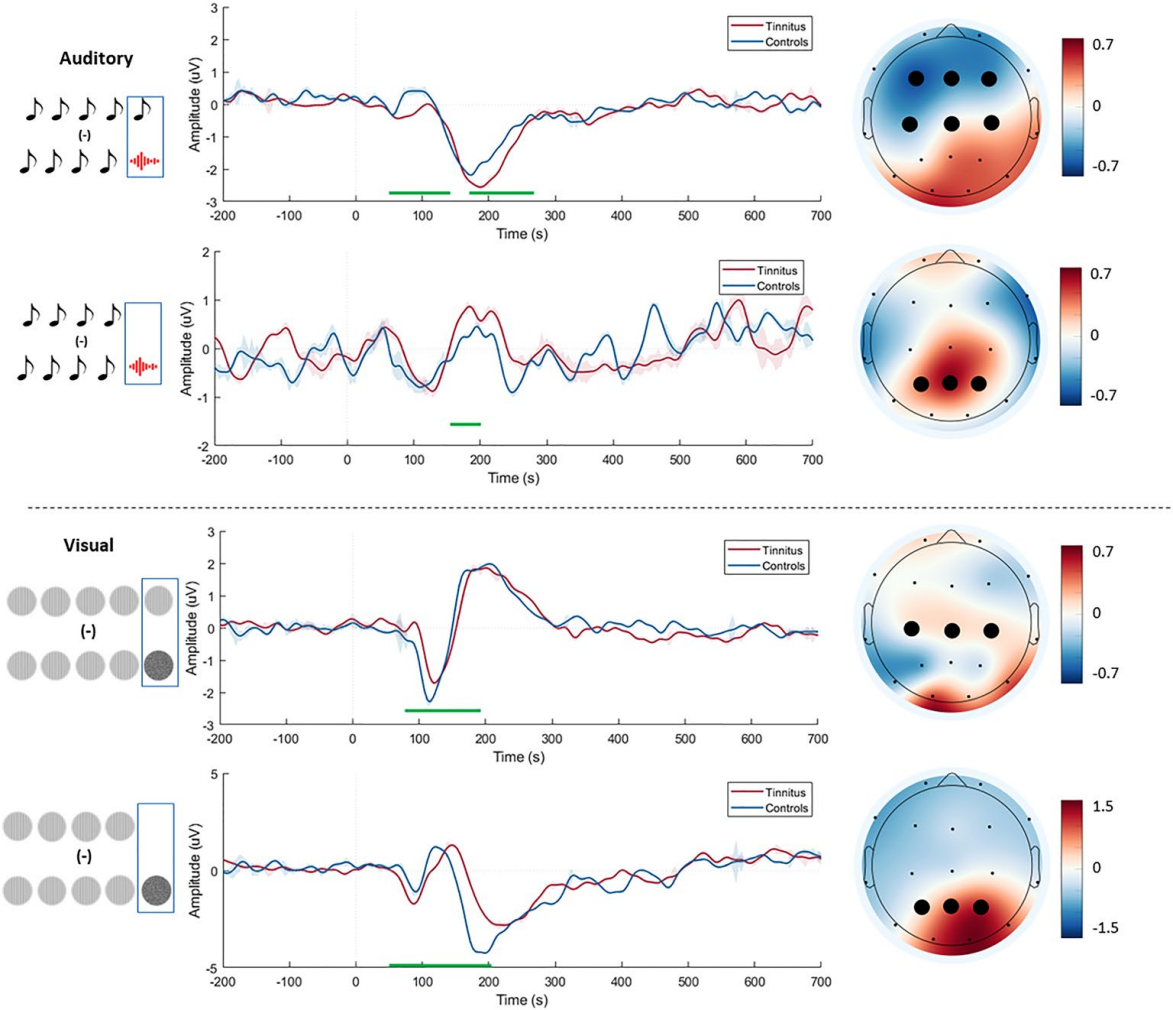
Scalp maps and event-related potentials for the prediction error generated by the subtraction of the responses of the two deviants and the standard in the MMN timeframe. Scalp maps show the mean amplitude in the mis-match negativity time frame for the difference between the two groups in the auditory (upper section) and visual (lower sections) domains. The event-related potentials show the prediction error response of the controls (blue) and tinnitus (red) group and the significant difference between them (green line). The “0” time point corresponds to the onset of the 5th stimulus in the sequence. The electrodes in BOLD show the electrodes that show a significant difference at all time points indicated by the green line. The red and blue shading around the lines represent the standard error bars of the prediction error signal.

In the auditory domain, we observe a significant difference between tinnitus and control groups in the MMN (140–300 ms) time frame. Particularly, we observe an increase in negative polarity during MMN time frame. The cluster of electrodes showing significant difference are shown in BOLD on row 1 of Fig. 3 with the corresponding ERP showing the average ERP from these electrodes with the significant time points. The channel-time point clusters that were significant were found in 92.5% of the comparison maps at *p* < 0.05 at a cluster level.

In the visual domain, we also observe a significant difference between the tinnitus and control groups in the MMN (88–200 ms) time frame. From a topographical view, we observe a decrease in amplitude of prediction error signal in the MMN timeframe for the tinnitus group in the central electrodes. The cluster of electrodes showing these changes are signified in BOLD. These changes are reflected in row 3 of Fig. 3, with the corresponding ERP showing the average ERP from these electrodes with the significant time points. The channel-time point clusters that were significant were found in 92.5% of the comparison maps at *p* < 0.05 at a cluster level.

In both the auditory and visual domain we do not observe a reliable change in P300 amplitudes.

### Stimulus-driven prediction error: difference between unexpected omission and standard stimulus sequences

In the auditory domain, we observe a significant difference between the two groups in the MMN (160–192 ms) time frame. Particularly, we observe an increased positivity in the parietal-occipital electrodes. The cluster of electrodes showing these changes are signified in BOLD. These changes are reflected in row 2 of Fig. 3, with the corresponding ERP showing the average ERP from these electrodes with the significant time points. The channel-time point clusters that were significant were found in 92.5% of the comparison maps at *p* < 0.05 at a cluster level.

In the visual domain, we see a pattern similar to the auditory domain with significant difference between the two groups in MMN (76–212 ms) timeframe. We observe a decreased amplitude in the MMN timeframe in the parietal electrodes, unlike in the auditory domain. These changes are evident in the respective ERPs. The cluster of electrodes showing these changes are signified in BOLD. These changes are reflected in row 4 in Fig. 3, with the corresponding ERP showing the average ERP from these electrodes with the significant time points. The channel-time point clusters that were significant were found in 92.5% of the comparison maps at *p* < 0.05 at a cluster level.

We observed no significant differences between the control and tinnitus groups in the P300 time frame. We also did not observe a significant difference in the context-driven prediction errors between the two groups.

### Post-hoc correlations with behavioural data

The results of the bivariate correlation between the amplitude of the different prediction errors signals at different timeframes and the behavioural variables are summarised in Table 1. Importantly we observe a significant correlation between THI score and the MMN amplitude of the sensory prediction error in the auditory domain.

**Table 1:** Results of bivariate correlation between amplitudes of different prediction error conditions and the behavioural variables.

Prediction error condition	Age (years) (all participants)		Mean hearing loss (dB) (all participants)		VAS for loudness (cm) (tinnitus)		Duration (years) (tinnitus)		Tinnitus Handicap Inventory (tinnitus)	
	ρ-value	*p*-value	ρ-value	*p*-value	ρ-value	*p*-value	ρ-value	*p*-value	ρ-value	*p*-value
Auditory domain										
Deviant—Standard (MMN)	0.34	0.075	0.12	0.531	− 0.29	0.224	0.13	0.636	− 0.22	0.404
Unexpected omission—Standard (MMN)	− 0.17	0.378	− 0.08	0.689	0.24	0.368	− 0.28	0.288	*0.65*	*0.007*
Visual domain										
Deviant—Standard (MMN)	0.12	0.532	0.06	0.764	− 0.07	0.799	− 0.09	0.736	− 0.15	0.571
Unexpected omission–Standard (MMN)	0.37	0.046	0.33	0.088	0.14	0.613	0.14	0.137	0.20	0.450

Significant values after multiple comparisons are in [italics].

## Discussion

In the current study we use tinnitus as a model to investigate if phantom perception is a result of aberrant predictive processing only in their respective deafferented sensory domains or whether it reflects an anomaly in higher-order systemic predictive processing which extends to other sensory domains as well. We probe the predictive coding system using auditory and visual stimuli designed using the well-established local–global oddball paradigm^23^ with three stimulus sequences (standard, deviant, omission). Subtracting the responses of frequent (standard, expected omission) from the rare (deviant, unexpected omission) sequences, we get two kinds of prediction errors—sensory and contextual. Sensory prediction errors characterise a deviance based on changing the stimulus characteristics of the incoming information, whereas contextual prediction errors characterise a deviance based on changing the expectation of the same incoming information. We demonstrate that tinnitus primarily affects processing of sensory prediction errors in the deafferented sensory domain (auditory) and a domain independent of it (visual).

### Effect of tinnitus on auditory prediction errors

Previous research in tinnitus about changes in prediction errors was solely conducted in the auditory domain. Most work surrounded the examination of the MMN and these results were inconsistent across studies showing increased^37^, decreased^38,39^ or no changes^40^ in MMN amplitude in the tinnitus group when tones were presented at frequencies distant from that of their hearing loss (similar to the current study). In schizophrenia, however, research showed a consistent and robust decrease in MMN amplitude^41,42^. Furthermore, studies in patients with hallucinations reveal decreased MMN amplitude compared to non-hallucinating patients and healthy controls^42^.

In the auditory and visual domain, the pattern of sensitivity to sensory prediction errors in the tinnitus group depends on the domain itself. In the auditory domain, we observe that people with tinnitus have an increased prediction error as seen as an increase in MMN amplitude in both the sensory prediction error conditions, however, in the visual domain, we observe a decrease in MMN amplitude. The MMN is a pre-attentive ERP component that records the automatic change in stimulus characteristics^43^. The higher deviance in the auditory domain is consistent with our previous study^20^ and with the idea of increased salience^44^ and attention^45^ in the auditory domain attributed to the presence of tinnitus. It could also be perceived as a compensation for the auditory deafferentation where the tinnitus is “filling in the missing information” by acting as a maladaptive compensation as previously hypothesised^3^.

### Effect of tinnitus on visual prediction errors

The effect of tinnitus on visual processing is very under-researched although there is abundant literature showing cross-modal connection between the auditory and visual pathways^46–48^. Patients who have undergone removal of vestibular schwannoma experience a rare form of gaze-evoked tinnitus showing the relationship between audio-visual pathways in a subset of people with tinnitus^49^. Additionally, preliminary evidence shows a decrease in speed of visual processing in people with tinnitus with a decline in detection and encoding of different visual stimuli^50^. Furthermore, task-related deactivation of the visual cortex was decreased in patients with tinnitus^51^.

In the current study, we observe a change in the predictive processing of visual stimuli in people with tinnitus. Interestingly, patients with tinnitus show an opposite effect in the visual domain compared to the auditory domain. Decrease in MMN amplitude in the visual domain suggests compromised signal detection and encoding as shown by a previous study. The MMN being a pre-attentive signal may also interpreted as “bottom-up attention” to stimulus-driven changes. A decrease in MMN in the visual domain in patients with tinnitus suggests changes in low-level visual processing. Further studies are required to elaborate the effect of tinnitus on visual processing.

### Implications for future research on tinnitus

Sensitivity to prediction errors not only in the sensory domain of the percept, but a domain independent of it provides evidence that changes in the predictive coding system are domain-general involving higher-order regions. Even if one argues that the changes in stimulus-driven prediction errors between the two groups in the auditory domain may be attributed to hearing loss, a similar pattern of activation observed in the visual domain, where changes to the visual field is corrected with spectacles. Furthermore, previous research argues that changes in the brain response to the omission of a stimulus can only be explained by predictive coding and not by neural adaptation theories^23^. Therefore, these changes in both sensory prediction errors in the auditory and visual domain may be directly attributed to aberrations in a higher-order predictive coding system orchestrated by the presence of tinnitus.

This is also seen in the significant positive correlation between the auditory prediction error and THI score. The parietal electrodes that encode this prediction error overlay regions that have been previously implicated in tinnitus-related distress^52,53^ (i.e. posterior cingulate cortex, PCC) and a primary hub in the auditory prediction error^20^. Furthermore, the increase in surprise to a missing auditory stimulus in tinnitus group also signals a change in context in addition to the change in input stimulus characteristics. The correlation of this change in context to subjective distress is in line with the idea that patients with tinnitus are more likely process non-threatening stimuli, or in this case the unexpected missing of sensory information, in a distressing context^54^. Additionally, we know from previous research that there is an increased connectivity between the auditory and distress networks alluding to this change in contextual processing of stimuli in tinnitus^55–57^.

However, we do not observe a significant difference in the prediction error in the P300 timeframe in both stimulus-driven prediction error conditions nor in purely context-driven prediction errors. This result differing from the previous study may be because of low trial count—given that this is a passive oddball paradigm, the P300 component can be noisy. Additionally the context-driven prediction error consists of only 30 trials which can make the differences in this condition also quite noisy. Furthermore, it is also important to keep in mind that the patient group is different compared to the previous study, where the current study includes people with hearing loss, who were excluded in the previous study. Future studies could therefore look into the effect of hearing loss on prediction error components using the local–global auditory paradigm and its impact on tinnitus.

### Limitations and future directions

The current study provides empirical evidence to phantom percepts being a systemic disorder rather than simply an auditory processing disorder. However, like any other study, it comes with some limitations. The first is the low number of trials in the unexpected omission condition. The reason to decrease this number in the current study was to implement a pilot study in a clinical setting. In order to manage the clinic proceedings with the time taken for the study, this decision was made as a compromise. The low sample size could be another potential limitation. It is important to keep in mind that this is a pilot study investigating the consequence of tinnitus in visual and auditory predictive processing. Secondly, it is important to keep in mind that increase and decrease in sensitivity to auditory and visual ERP components explains the positioning of the dipoles. Even though at a scalp level there may be a negative polarity at some electrodes, the source level information might be different. Therefore, it would be beneficial to perform this experiment inside an MRI scanner to better grasp the source-level changes of these scalp-level potentials. Although there is no correlation of the amplitude of the prediction error with mean hearing loss, future research can use mean hearing loss and individual hearing thresholds at different frequencies to account for any differences in the results based on hearing loss.

## Conclusion

From the current study, we therefore see that phantom percepts not only reflect changes in predictive coding in their own sensory domains but transcend to other sensory domains. Particularly patients with an auditory phantom perception are more sensitive to prediction errors in the auditory domain and less sensitive to prediction errors in the visual domain. Furthermore, the auditory prediction error is correlated to tinnitus-related distress. The fact that tinnitus affects visual processing not only reinforces the cross-modal plasticity between auditory and visual domains but also provides preliminary evidence that phantom percepts are not simply an auditory predictive processing disorder but possibly a symptom of a more systemic change in the higher-order systems-level predictive coding mechanism transcending the auditory domain.

## Data Availability

The data and code are present with the corresponding author. They will be available on email request. Interested members may email the corresponding author. The anonymised data will be provided after removing the identifying personal information. The code will also be distributed on request with the corresponding author. The datasets generated during and/or analysed during the current study are available from the corresponding author on reasonable request via email.
